# Application and evaluation of hydrodissection in microwave ablation of liver tumours in difficult locations

**DOI:** 10.3389/fonc.2023.1298757

**Published:** 2023-11-16

**Authors:** Yuan Song, Meng Wu, Ruhai Zhou, Ping Zhao, Dan Mao

**Affiliations:** Department of Ultrasound, The Affiliated People’s Hospital of Ningbo University, Ningbo, Zhejiang, China

**Keywords:** hepatocellular carcinoma, hydrodissection, microwave ablation, difficult location, ultrasound

## Abstract

**Objective:**

To investigate the safety and mid-term outcomes of hydrodissection-assisted microwave ablation (MWA) of hepatocellular carcinoma (HCC) in various difficult locations.

**Methods:**

A total of 131 HCC patients who underwent ultrasound-guided MWA from March 2017 to March 2019 were included. Following ultrasound examination, patients with tumors at difficult locations were treated with hydrodissection-assisted MWA (hydrodissection group), while those with tumors at conventional locations received MWA (control group). Both groups were compared concerning baseline characteristics, ablation parameters, complete ablation rates, and complication rates. Kaplan-Meier curves analyzed local tumor progression and overall survival, with stratified analysis for different difficult locations (adjacent to gastrointestinal tract, diaphragm, and subcapsular tumors). Additionally, Cox regression analyses were conducted to assess the impact of different difficult locations on these outcomes.

**Results:**

Complete ablation rates were similar between the hydrodissection and control groups (91.4% *vs.* 95.2%, P>0.05). Postoperative complications occurred in three patients, including liver abscess and biliary injury. No significant differences in major or minor complication rates were found between the groups (P>0.05). Local tumor progression was detected in 11 patients (8.4%) at the end of the follow-up period. Neither cumulative local tumor progression rate (P=0.757) nor overall survival rate (P=0.468) differed significantly between the groups. Stratified analysis showed no effect of tumor location difficulty on cumulative local tumor progression or overall survival. Tumor number and size served as independent predictors for overall survival, while minimal ablation margin ≤ 5mm independently predicted local tumor progression. In contrast, the tumor location was not statistically significant. Sensitivity analyses corroborated the robustness of the models.

**Conclusion:**

Hydrodissection-assisted MWA for HCC in various difficult locations demonstrated safe and effective, with complete ablation and mid-term outcomes comparable to those for tumors in conventional locations.

## Introduction

Hepatocellular carcinoma (HCC), the sixth most common global neoplasia, is associated with a remarkably high mortality rate. Liver transplantation and surgical resection have been established as the gold-standard therapeutic approaches for hepatic tumors. However, over 75% of patients are precluded from surgery due to inadequate hepatic functional reserve, multifocality, advanced disease, or comorbidities (1, 2). Ablation therapy, particularly microwave ablation (MWA), has thus become an alternative for certain HCC patients, displaying benefits such as a wider ablation zone, shorter duration, and less heat-sink effect, favoring tumors over 3 cm or near major vessels (3–5).

Despite the evident advantages of MWA, previous studies have shown inferior outcomes in primary liver cancer cases involving difficult locations when treated with MWA (6, 7). In an effort to circumvent thermal damage to neighboring organs, ablations near the gastrointestinal tract, diaphragm, gallbladder, and kidneys frequently fail to achieve comprehensive treatment, thereby increasing the risk of residual malignancy and locoregional metastasis. Additionally, the incidence of complications such as intraperitoneal bleeding, gastrointestinal injury, and tract seeding tends to be higher in these areas (8, 9).

Recent studies have shown that hydrodissection can physically separate tumor lesions from adjacent tissues in patients with liver tumors in difficult locations, achieving optimal ablation margins and protecting nearby organs (10, 11). Hydrodissection is an established thermal protection method in percutaneous thermal ablation. It involves the injection of fluid between the lesion and adjacent vital structures, which reduces the risk of thermal damage and minimizes postoperative complications. Studies conducted by Xiaoyin et al. (12) have validated the safety and efficacy of hydrodissection-assisted MWA in the treatment of thyroid nodules, emphasizing its utility when anatomical structures are closely intertwined. In the context of liver tumors, particularly those located adjacent to vital structures, hydrodissection has been similarly recognized for its significance. Garnon et al. (13) illustrated the application of this technique in percutaneous thermal ablation of sub-cardiac hepatic tumors, demonstrating its pivotal role in enhancing procedural safety by maintaining a protective barrier between the ablation zone and adjacent vital tissues.

Although some studies have evaluated hydrodissection in hepatic malignancy ablation, most have focused on a particular challenging position, with limited analysis of effectiveness across various difficult locations or the impact on patient prognosis (14–16). Therefore, this study retrospectively analyzed the clinical data of patients with liver tumors in different difficult locations treated with hydrodissection-assisted MWA, and compared it with patients with liver tumors in conventional locations. The aim was to investigate the effectiveness and mid-term outcomes of ablation in various difficult tumor locations.

## Materials and methods

### Patients

This retrospective analysis included patients who received thermal ablation for HCC from March 2017 to March 2019. All patients had a confirmed diagnosis of HCC by the combination of radiological and pathological criteria. Inclusion criteria were as follows (1): patients who were either not suitable for or refused hepatic resection, with either solitary tumors ≤7cm or multiple tumors (up to 3) ≤3cm in maximum diameter (2); absence of tumor thrombus in the main portal or inferior vena cava (3); hepatic function status of Child-Pugh Class A or B (4); ultrasound revealed the presence of an appropriate route for puncture and ablation. Exclusion criteria included (1): extrahepatic diseases or distant metastasis (2); platelet count less than 50×10^9^/L, with uncorrectable coagulation dysfunction (3); incomplete patient data. Tumors in difficult locations are defined as those where at least one tumor is located less than 5mm from the liver capsule, diaphragm, or gastrointestinal tract as confirmed by contrast-enhanced CT or MRI. Ethical approval for this study was granted by the ethics review committee of the institution (YY2023035). Informed consent was waived owing to the retrospective nature of this research.

### Clinical baseline data

Demographic data and clinical parameters, including age, gender, Child-Pugh classification, presence or absence of cirrhosis, previous treatments, tumor dimensions, tumor number, and Alpha-fetoprotein (AFP) levels, were collected by reviewing electronic medical records.

### Preoperative examination

**Figure 1:**
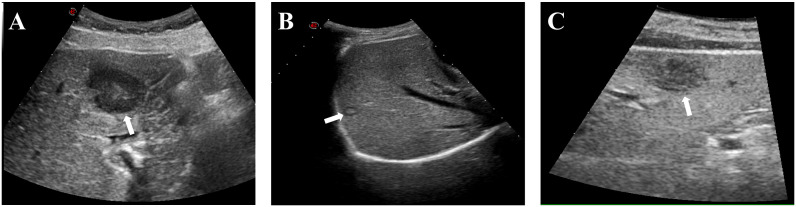
Typical ultrasound images of patients with liver tumors at difficult locations. **(A)** Liver tumor adjacent to the gastrointestinal tract (white arrow); **(B)** Liver tumor adjacent to the diaphragm (white arrow); **(C)** Liver subcapsular tumor (white arrow).

A full clinical assessment was performed, which encompassed complete blood count, clotting analysis, hepatic and renal function, and serum tumor markers. Ultrasound was used to assess the lesion site, size, number, and relation to important structures. Additionally, the distribution of blood flow in and around the tumor was observed to determine the puncture pathway of the MWA needle. The selection of the anesthesia type was dependent on the location, number, and size of the tumor. Ablation strategies were decided by three interventional radiologists with over 10 years of experience. Ultrasound images of liver tumors in difficult locations are shown in **Figure 1**.

### Hydrodissection technique

Under the guidance of ultrasound (Philips EPIQ 7), an 18-G PTC puncture needle (Hakko, Tokyo, Japan) was inserted between the liver capsule and parietal peritoneum or adjacent structures, and the needle core was then removed. A small amount of 0.9% saline was gradually infused through the cannula to separate the liver from the surrounding tissue. When injection resistance was absent and ultrasound indicated clear separation, the guidewire was inserted and the puncture needle was withdrawn. The catheter sheath was subsequently introduced over the guidewire, and a continuous 0.9% saline infusion was maintained through a connected infusion system until successful separation was confirmed (>0.5cm between tumor and surrounding structures).

### Ablation procedures

**Figure 2:**
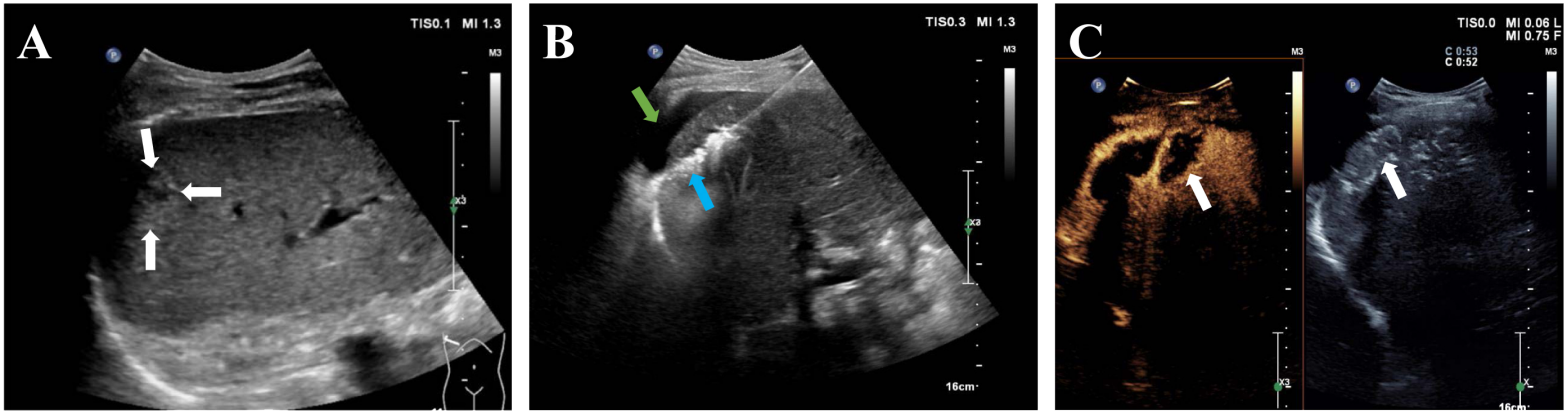
A 68-year-old man with hepatocellular carcinoma before and after MWA. **(A)** Ultrasound image showed a 1.6-cm hepatocellular carcinoma in the subcapsular region of the liver (white arrow).; **(B)** After the saline injection hydrodissection (green arrow), the electrode needle was inserted into the tumor under ultrasound guidance (blue arrow); **(C)** Postoperative CEUS showed no enhancement in the whole ablation area (white arrow).

Percutaneous MWA procedures were performed under general anesthesia. All patients underwent ablation using a MWA system (KY-2000, Kangyou Medical Instruments, Nanjing, China), equipped with a 2450 MHz microwave generator and a 15G water-cooling ablation needle. Based on preoperative planning, the 18G electrode needle was inserted into the tumor via ultrasound guidance. Puncture routes for tumors in conventional locations were designed to avoid lungs, large blood vessels, gallbladder, and other organs. Depending on tumor size, ablation was either single-point (for diameters ≤3cm) or multi-point (for diameters >3cm), executed at 40-60 W for a duration of 3-8 minutes. The ablation was deemed complete when the high-echoic area under ultrasound covered the entire tumor volume and an additional 0.5 cm of adjacent hepatic parenchyma. If the ablation area was found insufficient, the needle was repositioned. For tumors in difficult locations, hydrodissection was implemented during ablation. The hyper-echogenicity areas induced by ablation in tumors near the liver surface were continuously monitored with ultrasound, and the needle depth was adjusted if the area exceeded the liver capsule to prevent repeat ablation at the same puncture site. Contrast-enhanced ultrasound (CEUS) was performed immediately after MWA to evaluate the ablation area (**Figure 2**). For tumors adjacent to the gastrointestinal tract, the needle trajectory was designed to be parallel or distant from visceral organs as much as possible. Post-operatively, these patients were subjected to a 48-hour fast and were administered antacids, antibiotics, and somatostatin to reduce the risk of complications such as gallbladder or gastric perforation.

### Follow-up and effectiveness assessment

Perioperative and follow-up evaluations were conducted on patients, and the ablation parameters, complete ablation rate, complication incidence, local tumor progression, and overall survival were analyzed in the two groups. The initial follow-up was scheduled one month after the MWA, during which coagulation parameters, serum tumor markers, and liver function were reassessed. Meanwhile, ablation margins were assessed based on contrast-enhanced CT scans conducted preoperatively and one month postoperatively. The largest diameter of the non-contrast-enhancing zone was measured in axial, coronal, or sagittal planes one month after the operation. For ablation margins, distances to adjacent anatomical landmarks were documented on both sets of scans. The margin at each landmark was determined by subtracting the preoperative from the postoperative distance, based on a method described by Wang et al. (17). The smallest resulting value was deemed the minimal ablation margin. Subsequent follow-ups were scheduled every three months with imaging and serum assessments. Complete ablation was defined as the absence of enhancement within the ablation zone on contrast-enhanced CT, MRI, or CEUS one month post-operation (18). Patients with complete ablation underwent subsequent follow-ups to evaluate the rate of local tumor progression. Residual tumors post-ablation (characterized by irregular enhancement around the ablation lesion) were treated with a secondary ablation or interventional embolization. Major complications were life-threatening, resulted in significant morbidity and disability, or prolonged hospital stay (19). Minor complications were self-limiting, necessitating no additional treatment. Local tumor progression was identified as the appearance of tumor foci at the edge of a sufficiently ablated zone, confirmed at least once through contrast-enhanced imaging during the post-procedure follow-up period (20). Overall survival was calculated from the day of ablation to the date of death or the final follow-up.

### Statistical analysis

Statistical analyses were conducted using SPSS 22.0 software (IBM, NY, USA) and Medcalc 15.2 software (MedCalc, Ostend, Belgium). Normal distribution measurements were expressed as mean ± standard deviation, while skewed distributions were presented as median (range). Comparisons between groups were performed by independent samples t-test or the Mann-Whitney test. Categorical data were expressed as a number (percentage), and the chi-square test or Fisher’s exact test was used to compare the data between the two groups. The Kaplan-Meier curves were plotted to assess local tumor progression and cumulative survival rates in both groups, and stratified analysis was conducted for tumors in different difficult locations. Univariate and multivariate Cox regression analyses were further performed to evaluate the impact of difficult tumor location on local tumor progression and overall survival, corroborated by Bootstrap resampling with 1000 replicates for sensitivity analysis. A p-value less than 0.05 was considered statistically significant.

## Results

### Comparison of clinical data

**Figure 3:**
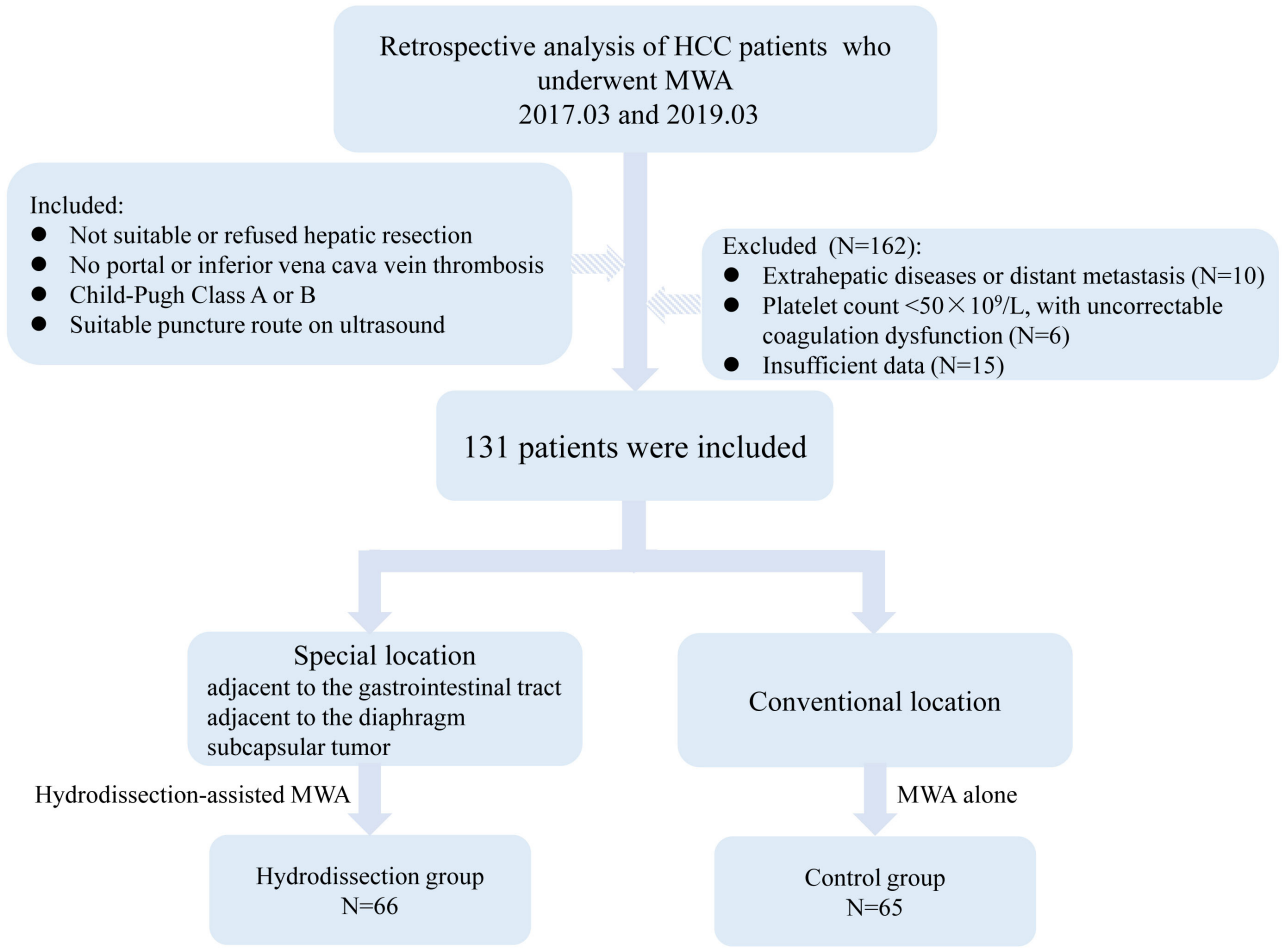
Flow diagram of the included patients.

Based on the usage of the hydrodissection during MWA, patients were categorized into hydrodissection group and control group. Sixty-six patients with tumors in difficult locations underwent hydrodissection-assisted MWA, including 28 cases (42.4%) near the gastrointestinal tract, 21 cases (31.8%) near the diaphragm, and 17 cases (25.8%) as subcapsular liver tumors. The remaining 65 patients with conventional tumors received MWA and served as the control group. The patient selection flowchart was shown in **Figure 3**.

The baseline characteristics of the included patients were displayed in **Table 1**. A total of 131 patients (176 lesions) underwent MWA, with an average age of 58.6 ± 9.8 years. Most patients were male, rated as Child-Pugh grade A, with histories of hepatitis virus infection. In the hydrodissection group, 74 out of 93 lesions were treated with hydrodissection-assisted MWA, while the remaining 19 lesions received MWA alone. In the control group, all 83 lesions were subjected to MWA. Among the hydrodissection group, 18 individuals (27.3%) had multiple lesions, and the maximum tumor diameter exceeded 3 cm in 25 patients (37.9%). In the control group, 15 patients (23.1%) presented with multiple lesions, and 22 patients (33.9%) had a maximum tumor diameter >3 cm. No statistically significant difference was observed in the baseline characteristics between the two groups (P>0.05).

**Table 1:** Demographic data and tumor characteristics of the two groups.

	Hydrodissection group	Control group	P
Patients	66	65	
Age, years	58.2 ± 11.0	59.1 ± 8.5	0.597
Gender, n(%)			
Male	47(71.2)	43(66.2)	0.575
Female	19(28.8)	22(33.8)	
History of hepatitis virus infection, n(%)			
Yes	52(78.8)	48(73.8)	0.543
No	14(21.2)	17(26.2)	
Liver cirrhosis, n(%)			
Yes	38(57.6)	42(64.6)	0.475
No	28(42.4)	23(35.4)	
Tumor number, n(%)			
Single	48(72.7)	50(76.9)	0.688
Multiple	18(27.3)	15(23.1)	
Child-Pugh class, n(%)			
A	55(83.3)	53(81.5)	0.822
B	11(16.7)	12(18.5)	
Tumor size, n(%)			
≤3 cm	41(62.1)	43(66.2)	0.886
3~5 cm	19(28.8)	17(26.2)	
>5 cm	6(9.1)	5(7.7)	
AFP level, n(%)			
Positive	50(75.8)	46(70.8)	0.558
Negative	16(24.2)	19(29.2)	
History of intervention, n(%)			
Yes	20(30.3)	18(27.7)	0.848
No	46(69.7)	47(72.3)	
History of hepatectomy, n(%)			
Yes	6(9.1)	9(13.8)	0.425
No	60(90.9)	56(86.2)	

AFP, Alpha-fetoprotein.

### Ablation parameters and outcomes

In this study, 131 patients underwent a total of 140 ablation procedures, with a technical success rate of 100%, and the average number of treatments was 1.5 ± 0.51. Single ablation treatment was performed on 119 patients (90.8%), while 12 patients (9.2%) required repeated ablation. In the 74 lesions treated with hydrodissection-assisted MWA, saline injection separation was successful in 100% of cases, with an average fluid volume of 723.8 ± 240.5 ml. Although the hydrodissection group experienced a slight increase in ablation time, ablation frequency, and ablation duration compared to the control group, these differences were not statistically significant(P>0.05). No significant difference was observed between the hydrodissection group and the control group in terms of the number of antenna insertions (P>0.05). Initial follow-up revealed that the average ablation zone sizes in the control group were (4.62 ± 0.86) cm, compared to (4.60 ± 0.72) cm in the hydrodissection group (P>0.05). The hydrodissection group showed similar ablation zone sizes across tumor locations adjacent to the gastrointestinal tract (4.67 ± 0.62 cm), adjacent to the diaphragm (4.36 ± 0.76 cm), and subcapsular tumors (4.78 ± 0.74 cm), with no statistical significance among these subgroups (P>0.05). The proportion of patients with a minimal ablation margin of ≤5 mm was 32.3% in the control group and 41.1% in the hydrodissection group. Within the hydrodissection group, the proportions were 42.9% for tumors adjacent to the gastrointestinal tract, 42.9% for those adjacent to the diaphragm, and 35.3% for subcapsular tumors. These differences were not statistically significant (P>0.05). Data from the hydrodissection group indicated a complete ablation rate of 91.4% in the hydrodissection group, which was similar to the control group, with no statistical significance (P>0.05). Further analysis demonstrated that the complete ablation rates for lesions adjacent to the gastrointestinal tract (92.3%), adjacent to the diaphragm (90.0%), and subcapsular liver tumors (91.7%) were not statistically different compared to the control group (P>0.05). Moreover, there was no statistical significance in the differences among the three categories (P>0.05), as shown in **Table 2**.

**Table 2:** Outcome of MWA in the hydrodissection and control group.

Variates	No. of patients	Ablation time (min)	Power (watts)	No. of ablation sessions	No. insertions	Ablation zone size (cm)	Minimal ablation margin ≤5 mm (%)	Complete ablation(%)
Control group	65	10.9 ± 3.56	51.5 ± 2.35	1.4 ± 0.49	2.4 ± 1.13	4.62 ± 0.86	32.3	95.2
Hydrodissection group	66	13.6 ± 4.27	53.4 ± 2.91	1.7 ± 0.50	2.5 ± 1.19	4.60 ± 0.72	41.1	91.4
Adjacent to the gastrointestinal tract	28	13.6 ± 4.27	52.9 ± 2.91	1.6 ± 0.50	2.6 ± 1.19	4.67 ± 0.62	42.9	92.3
Adjacent to the diaphragm	21	12.9 ± 4.29	53.6 ± 2.93	1.8 ± 0.51	2.5 ± 1.20	4.36 ± 0.76	42.9	90.0
Subcapsular tumor	17	14.6 ± 4.31	53.8 ± 2.94	1.6 ± 0.51	2.2 ± 1.22	4.78 ± 0.74	35.3	91.7

MWA, microwave ablation.

### Post-ablation complications

Severe complications occurred in 3 of 131 patients (2.3%), comprising two hepatic abscesses and one biliary injury. These patients improved after percutaneous drainage and anti-infection treatment. The major complication rate was 3.0% for the hydrodissection group and 1.5% for the control group, with no significant difference in hepatic abscess or biliary injury (all P>0.05). Minor complications included abdominal pain, fever, gastrointestinal symptoms, minimal pleural effusion in one patient, transient hepatic function abnormality in three patients, and two asymptomatic bilomas, all of which experienced rapid remission after treatment. There was no statistically significant difference in the incidence of various minor complications between the two groups (all P>0.05). Details of the major and minor complications were presented in **Table 3**.

**Table 3:** Comparison of complication rate between hydrodissection and control group.

	Hydrodissection group	Control group	P
Patients	66	65	—
Major complications, *n*(%)			
Liver abscess	2 (3.0)	0	0.496
Biliary injury	0	1 (1.5)	0.496
Minor complications, *n*(%)			
Abdominal pain	7 (10.6)	5 (7.7)	0.763
Fever	6 (9.1)	4 (6.2)	0.527
Gastrointestinal symptoms	3 (4.5)	5 (7.7)	0.492
Minimal pleural effusion	0	1 (1.5)	0.496
Transient liver dysfunction	1 (1.5)	2 (3.1)	0.619
Asymptomatic biloma	0	2 (3.1)	0.244

### Local tumour progression and survival

**Figure 4:**
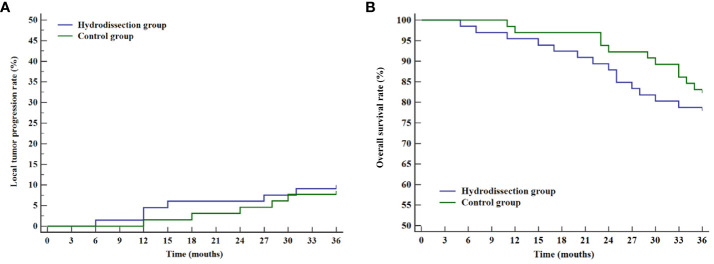
Curves of cumulative local tumor progression rates and overall survival rates of the two groups. **(A)** Kaplan-Meier curve of cumulative local tumor progression rates; **(B)** Kaplan-Meier curve of overall survival rates.

Patients with complete ablation were followed for 36 months to assess local tumor progression rates and overall survival in both groups. Local tumor progression was observed in 11 patients (8.4%), including 6 in the hydrodissection group and 5 in the control group. The 1-, 2-, and 3-year cumulative local tumor progression rates for the hydrodissection group were 3.0%, 6.1%, and 9.1%, respectively, compared to 1.5%, 4.6%, and 7.7% for the control group, with no significant difference (P=0.757, **Figure 4A**). The 1-, 2-, and 3-year overall survival rates for the hydrodissection group were 95.5%, 87.9%, and 78.8%, respectively, while those for the control group were 96.9%, 92.3%, and 83.1%, showing no statistical significance between the two groups (P=0.468, **Figure 4B**).

### Stratified analysis of difficult locations

**Figure 5:**
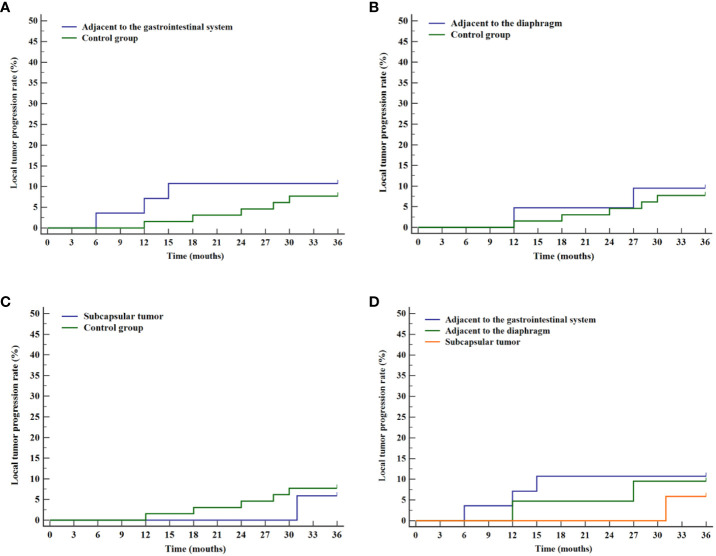
Curves of cumulative local tumor progression for various difficult locations and the control group. **(A)** Comparison of cumulative local tumor progression rates between tumor adjacent to the gastrointestinal system and the control group. **(B)** Comparison of cumulative local tumor progression rates between tumor adjacent to the diaphragm and the control group. **(C)** Comparison of cumulative local tumor progression rates between liver subcapsular tumor and the control group. **(D)** Comparison of cumulative local tumor progression rates for tumor in various difficult locations.

The prognosis of tumors in three difficult locations (adjacent to the gastrointestinal tract, adjacent to the diaphragm, and liver subcapsular) was assessed through stratified analysis of cumulative local tumor progression rates and overall survival rates. Kaplan-Meier survival curves revealed no statistical differences in cumulative local tumor progression rates for tumors adjacent to the gastrointestinal tract (P=0.596, **Figure 5A**), adjacent to the diaphragm (P=0.779, **Figure 5B**), and liver subcapsular tumors (P=0.778, **Figure 5C**), compared with the control group. Subsequent analysis for internal comparison among these locations showed no significant differences (P=0.843, **Figure 5D**).

**Figure 6:**
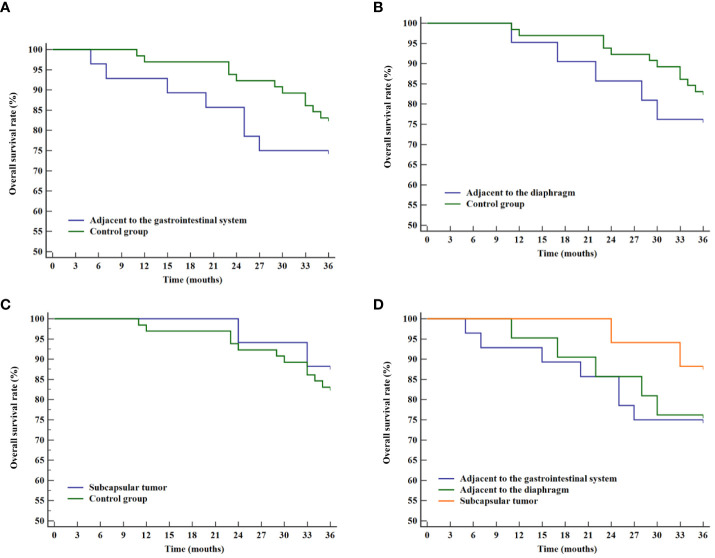
Overall survival curves for various difficult locations and the control group. **(A)** Comparison of overall survival rates between tumor adjacent to the gastrointestinal system and the control group. **(B)** Comparison of overall survival rates between tumor adjacent to the diaphragm and the control group. **(C)** Comparison of overall survival rates between liver subcapsular tumor and the control group. **(D)** Comparison of overall survival rates for tumor in various difficult locations.

Similarly, the analysis of overall survival rates revealed no statistical differences when compared with the control group for tumors adjacent to the gastrointestinal tract (P=0.297, **Figure 6A**), adjacent to the diaphragm (P=0.420, **Figure 6B**), and subcapsular tumors (P=0.598, **Figure 6C**). No statistical significance was found among the three difficult locations (P=0.516, **Figure 6D**).

### Univariate and multivariate analysis

Univariate Cox regression analysis (**Table 4**) revealed significant predictors for overall survival: tumor number (HR=3.066, P=0.009), Child-Pugh score (HR=4.025, P=0.001), tumor size (HR=4.845, P<0.001), and minimal ablation margin ≤ 5mm (HR=0.296, P=0.004). Age (HR=1.065, p=0.034) and minimal ablation margin ≤ 5mm (HR=0.142, P=0.003) were significant for local tumor progression. In the multivariate model (**Table 5**), tumor number (HR=3.268, P=0.024) and tumor size (HR=4.473, P=0.008) were independent factors affecting overall survival, while minimal ablation margin ≤5mm (HR=0.207, P=0.025) was an independent predictor of local tumor progression. However, the anatomical location of the tumor did not exhibit a statistically significant relationship with either overall survival or local tumor progression (P>0.05).

**Table 4:** Univariate analysis of factors associated with local tumor progression and overall survival.

Variables	Overall survival			Local tumor progression		
	HR	95%CI	P	HR	95%CI	P
Age	1.019	0.977-1.064	0.375	1.065	1.005-1.130	0.034
Gender (Male/Female)	0.777	0.306-1.971	0.595	0.648	0.178-2.353	0.509
Tumor location						
Adjacent to the gastrointestinal tract (Yes/No)	0.716	0.282-1.817	0.482	0.888	0.244-3.225	0.856
Adjacent to the diaphragm (Yes/No)	0.889	0.303-2.615	0.831	0.613	0.169-2.228	0.457
Subcapsular tumor (Yes/No)	0.934	0.278-3.144	0.912	0.521	0.143-1.893	0.322
Liver cirrhosis (Yes/No)	0.506	0.200-1.284	0.152	1.829	0.615-5.444	0.278
Tumor number (Single/Multiple)	3.066	1.326-7.092	0.009	2.738	0.895-8.372	0.077
Child-Pugh (A/B)	4.025	1.705-9.052	0.001	3.201	0.985-10.402	0.053
Tumor size (≤3cm/>3cm)	4.845	2.052-11.439	<0.001	2.662	0.895-7.921	0.078
AFP (Positive/Negative)	1.303	0.536-3.166	0.56	1.863	0.609-5.696	0.275
Ablation zone size	1.152	0.689-1.927	0.589	1.293	0.653-2.560	0.461
Minimal ablation margin ≤5mm (Yes/No)	0.296	0.128-0.684	0.004	0.142	0.039-0.515	0.003

**Table 5:** Multivariate analysis of factors associated with local tumor progression and overall survival.

Variables	Overall survival			Local tumor progression		
	HR	95%CI	P	HR	95%CI	P
Age	1.002	0.956-1.050	0.938	1.043	0.982-1.108	0.171
Tumor location						
Adjacent to the gastrointestinal tract (Yes/No)	0.413	0.136-1.256	0.119	0.721	0.158-3.292	0.673
Adjacent to the diaphragm (Yes/No)	0.414	0.111-1.543	0.189	0.42	0.083-2.113	0.293
Subcapsular tumor (Yes/No)	0.833	0.206-3.370	0.789	0.509	0.100-2.581	0.415
Tumor number (Single/Multiple)	3.268	1.277-8.365	0.024	2.433	0.661-8.947	0.181
Child-Pugh (A/B)	2.638	0.903-7.707	0.076	1.582	0.400-6.256	0.513
Tumor size (≤3cm/>3cm)	4.473	1.704-11.743	0.008	1.677	0.475-5.925	0.422
Ablation zone size	1.141	0.639-2.036	0.656	1.201	0.500-2.888	0.682
Minimal ablation margin ≤ 5mm (Yes/No)	0.527	0.207-1.341	0.179	0.207	0.052-0.819	0.025

Sensitivity analyses employing Bootstrap resampling techniques were executed to validate the multivariate Cox regression models for both overall survival and local tumor progression. These analyses confirmed the robustness of the models, indicating that all variables, including difficult locations, consistently maintained their respective roles in influencing both overall survival and local tumor progression.

## Discussion

With improvements in ablation technology, ultrasound-guided MWA in liver tumor treatment has become more prevalent, as supported by several studies that affirm its efficacy and safety. Successful ablation of liver tumors was found to be dependent on the ability of the ablation range to cover at least 5-10 mm of the lesion and its surrounding tissue, where an adequate ablation safety margin was correlated with a lower rate of local tumor progression (21, 22). However, the further application of MWA was limited by the incomplete ablation of some liver tumors due to insufficient safety distance with structures such as the diaphragm, gastrointestinal tract, and gallbladder. To minimize thermal damage to adjacent tissues during the treatment of liver tumors in difficult locations, the clinical use of hydrodissection technology to assist in ablation was initiated.

Despite studies affirming the utility of hydrodissection for liver tumors in difficult locations, few have explored mid-term clinical outcomes for multiple such tumors (23, 24). Recent studies by Li et al. (25) investigated liver tumors near the gastrointestinal tract, while Makovich et al. (26) focused on hepatocellular carcinoma ablation near vessels and below the diaphragm. In this study, a comparison was conducted between difficult and conventional locations liver tumors in terms of short-term effects and mid-term survival rates. Ultrasound-guided percutaneous MWA with hydrodissection was identified as a safe and effective treatment for hepatocellular carcinoma in difficult locations, achieving consistent local control across different positions.

Hydrodissection is an effective technique involving the use of saline to expand the extrahepatic space, thereby forming a thermal barrier between the ablation zone of the tumor and adjacent vital organs. This method not only facilitates the desired ablation effect on liver tumors that were previously challenging to fully eradicate but also minimizes unintentional thermal injury to nearby organs, reducing the incidence of postoperative complications (27). Several animal experiments have indicated that the application of hydrodissection can decrease damage to the diaphragm, stomach, and lungs, and substantially alleviate pain (28, 29). Similar results were reported by Park et al. (30), where patients with liver tumors experienced a significant reduction in pain within 24 hours following hydrodissection-assisted ablation, and the use of morphine during the perioperative period was also notably reduced.

Ultrasound-guided hydrodissection has been increasingly utilized in the ablation of liver, kidney, thyroid, and mediastinal tumors. Liu et al. (31) reported no significant difference in tumor progression or postoperative complications between hydrodissection-assisted MWA and MWA alone for subcapsular hepatocellular carcinoma and colorectal liver metastases. In another study, Cheng et al. (32) retrospectively evaluated the effective local control and renal protection in hydrodissection-assisted percutaneous MWA of renal cell carcinoma adjacent to the intestines. In treating various liver tumors in difficult locations, this study found a slight increase in ablation time, number and power, but no significant difference compared to conventional locations, with a rate exceeding 90%. A 100% isolation success rate indicated ease of operation and substantial clinical utility. Initial follow-up in the present study underscores that the minimal ablation margins were statistically comparable between the hydrodissection and control groups, even when tumors were located adjacent to critical structures like the gastrointestinal tract or diaphragm. These findings are in agreement with those reported by Kim et al. (33), further substantiating the utility of hydrodissection in hepatic ablation procedures. Moreover, the results revealed no significant difference in the complete ablation rates between liver tumors at various difficult locations, suggesting hydrodissection’s broad applicability to these tumors.

Consistent with findings by Johnson et al. (34), the present study found a low incidence of serious complications in HCC treated with MWA. Three patients encountered severe complications, namely liver abscess and biliary injury. The occurrence of the liver abscess was associated with factors such as tumor size and location, whereas biliary injury was related to thermal effects and changes in biliary blood supply. No statistical difference was detected in severe complication rates between hydrodissection and control groups, and no instances of intestinal or gallbladder perforation were observed, suggesting that hydrodissection-assisted MWA is a safe and feasible approach for liver tumors in difficult locations. The frequency of minor complications was higher, including abdominal pain and gastrointestinal symptoms, possibly linked to average tumor diameter and position. Increased body temperature may result from reabsorption of necrotic tissue, and patients near the diaphragm may experience abdominal and shoulder pain, while subcapsular tumors were more likely to cause hepatic region pain.

A comprehensive follow-up analysis was conducted to compare the mid-term outcomes of two groups of patients. The findings indicated no significant difference in cumulative local tumor progression rates or overall survival rates between the hydrodissection group and the control group during the follow-up period. In an initial study evaluating the efficacy of radiofrequency ablation in 138 HCC patients in high-risk locations, Hsieh et al. (35) reported a 2-year local tumor progression rate of 22.2% in those who did not undergo artificial ascites and pleural effusion instillation. Moreover, the study also found that the instillation of artificial fluids was associated with improved overall survival (HR=0.1, 95% CI: 0.01-0.95). The study suggested a poor prognosis for tumors in high-risk locations. In contrast, a retrospective study identified that hydrodissection-assisted ablation of liver tumors near the gastrointestinal tract resulted in a 2-year cumulative local progression rate of 5.7% (36). These findings suggest that the application of hydrodissection decreased local tumor progression in difficult locations, with outcomes similar to those in conventional locations. Stratification analysis of tumors in difficult locations (adjacent to the gastrointestinal tract, diaphragm, and under the liver capsule) revealed no significant variance in cumulative local tumor progression or overall survival rates compared to conventional locations, and no difference between these difficult locations. This highlights the feasibility and consistency of hydrodissection, regardless of tumor location, and broadens its potential therapeutic range. Cox regression analyses further identified prognostic factors affecting patient outcomes. Tumor number and size were independent determinants of overall survival, and minimal ablation margin ≤ 5mm significantly influenced local tumor progression. Notably, the anatomical location of the tumor was not statistically relevant for either outcome metric. Sensitivity analyses validated the robustness of these multivariate models, underscoring the reliability of these prognostic factors.

Nevertheless, this study has several limitations. The first pertains to the intricate relationship between difficult tumor locations and the therapeutic modalities employed, posing challenges for isolated analysis. Despite efforts to balance baseline characteristics, the intrinsic interdependence between these variables remains a confounding factor. To account for the variable deemed most clinically significant, difficult tumor locations were specifically incorporated into the Cox regression analyses as an independent variable. This methodological decision, while logical, does not fully resolve the limitations inherent in understanding the relationship between tumor location and treatment outcomes. The second limitation stems from the study’s retrospective design, which not only constrains the application of standard methods for calculating sample size in non-inferiority tests but also introduces the potential for selection bias and information bias. This may undermine the validity of the results and impact the robustness of the statistical analyses. While the chosen sample size was guided by previous literature and clinical experience, inherent limitations remain unaddressed. Third, the follow-up duration, being relatively brief, might overestimate both the rates of local tumor progression and overall survival. Additionally, the limited follow-up period may not capture late complications or the long-term efficacy of the hydrodissection technique. Therefore, future prospective studies with larger sample sizes and long follow-ups are essential to validate the effects of the hydrodissection technique in assisting microwave ablation in the treatment of hepatocellular carcinoma in difficult locations.

In conclusion, hydrodissection-assisted MWA offers a viable treatment option for HCC in difficult locations, demonstrating safety and mid-term outcomes comparable to those in patients with tumors in conventional locations. Tumor number and size were identified as independent predictors for overall survival, while a higher proportion of patients with a minimal ablation margin of ≤5mm were associated with local tumor progression. No statistically significant impact of tumor location on these outcomes was observed. Although these results are promising, additional research is required for a more comprehensive evaluation.

## Data availability statement

The original contributions presented in the study are included in the article/supplementary material. Further inquiries can be directed to the corresponding author.

## Ethics statement

The studies involving humans were approved by medical ethics committee of the Affiliated People’s Hospital of Ningbo University. The studies were conducted in accordance with the local legislation and institutional requirements. The participants provided their written informed consent to participate in this study.

## Author contributions

YS: Conceptualization, Formal Analysis, Methodology, Software, Writing – original draft, Writing – review & editing. MW: Funding acquisition, Investigation, Supervision, Validation, Writing – original draft, Writing – review & editing. RZ: Formal Analysis, Investigation, Methodology, Resources, Visualization, Writing – original draft. PZ: Data curation, Investigation, Methodology, Project administration, Resources, Software, Visualization, Writing – original draft. DM: Data curation, Formal Analysis, Resources, Software, Writing – original draft.
